# Uses, Tests for Purity, and Preparation of Chemical Re-agents

**Published:** 1891-11

**Authors:** 


Uses, Tests for Purity and Preparation of Chemical Re-agents, Employed in Qualitative, Quantitative, Volumetric, Dosimetric, Microscopic, and Petrographic Analysis, with a Supplement on the Use of the Spectroscope. By Chas. O. Curtman, M. D., Professor of Chemistry, and Director of Chemical Laboratory in the Missouri Medical College. Octavo, pp. 256, illustrated. John L. Borland, Book and Stationery Co. St. Louis, Mo.: 1890.

This volume will, no doubt, find a hearty welcome in the chemical world.

It contains a vast amount of information, of such a character as to save the busy professional man a great deal of work.

It is an excellent book, and a time saver; we congratulate the author on its production. J. A. M.

